# Sialic Acid Mimetic Microglial Sialic Acid-Binding Immunoglobulin-like Lectin Agonism: Potential to Restore Retinal Homeostasis and Regain Visual Function in Age-Related Macular Degeneration

**DOI:** 10.3390/ph16121735

**Published:** 2023-12-16

**Authors:** Michael J. Tolentino, Andrew J. Tolentino, Elizabeth M. Tolentino, Anitha Krishnan, Mohamed A. Genead

**Affiliations:** 1 Department of Ophthalmology, University of Central Florida College of Medicine, Orlando, FL 32827, USA; 2 Department of Ophthalmology, Orlando College of Osteopathic Medicine, Orlando, FL 34787, USA; 3 Aviceda Therapeutics, Cambridge, MA 02142, USA; akrishnan@avicedarx.com (A.K.); mgenead@avicedarx.com (M.A.G.); 4 Department of Biology, University of California Berkeley, Berkeley, CA 94720, USA; atolent@berkeley.edu; 5 Pratt Institute, Brooklyn, New York, NY 11205, USA; etolenti@pratt.edu

**Keywords:** microglia, macrophages, macular degeneration, sialic acid, Siglecs, nanoparticles, glycosylation, geographic atrophy, polysialic acid

## Abstract

Age-related macular degeneration (AMD), a leading cause of visual loss and dysfunction worldwide, is a disease initiated by genetic polymorphisms that impair the negative regulation of complement. Proteomic investigation points to altered glycosylation and loss of Siglec-mediated glyco-immune checkpoint parainflammatory and inflammatory homeostasis as the main determinant for the vision impairing complications of macular degeneration. The effect of altered glycosylation on microglial maintained retinal para-inflammatory homeostasis and eventual recruitment and polarization of peripheral blood monocyte-derived macrophages (PBMDMs) into the retina can explain the phenotypic variability seen in this clinically heterogenous disease. Restoring glyco-immune checkpoint control with a sialic acid mimetic agonist targeting microglial/macrophage Siglecs to regain retinal para-inflammatory and inflammatory homeostasis is a promising therapeutic that could halt the progression of and improve visual function in all stages of macular degeneration.

## 1. Age-Related Macular Degeneration (AMD)

### 1.1. Background

AMD is an age-related condition that progressively impairs central vision with increasing age. AMD affects the central portion of the retina called the macula, which is required for central vision and visually demanding tasks like recognizing faces, reading, and driving. Because central vision is important for these higher tasks, the impairment brought on by AMD results in decreased independence, mobility, and quality of life [1]. The potential for this form of vision loss is rated by surveyed individuals as one of the worst health outcomes possible [2].

AMD can be classified into early, intermediate, and late stages [3]. It is a disease that affects persons over the age of 60, and its incidence is increasing due to the rapid growth in the elderly population worldwide [4]. The prevalence of early- to intermediate-stage AMD in the US in 2019 reached approximately 18.34 million people [5]. This prevalence represents three times the number of patients with Alzheimer’s disease and is equal to all patients with a cancer diagnosis excluding melanoma [6,7]. By 2040, this disease that progressively causes central visual loss is estimated to affect 288 million people worldwide [8]. Currently, macular degeneration represents the third cause of vision loss secondary to ocular pathology worldwide [9]. Furthermore, the disease burden disproportionately affects less developed and low-income countries in Africa and Asia [10,11].

### 1.2. Clinical Presentation

The hallmark of early-stage AMD is the development of yellow subretinal deposits on the macula called drusen and/or abnormal pigmentary change called pigmentary mottling, clumping, or retinal pigment epithelial (RPE) change. When these changes worsen and reach a certain density, the stage is classified as intermediate. The late stage is reached when geographic atrophy (GA), neovascularization, or both develop [3].

From a visual function perspective, AMD can be divided into vision-threatening and non-vision-threatening stages. While drusen and RPE changes constitute the initiation of clinically detectable disease, these findings are usually not accompanied by any symptoms or noticeable vision loss [12]. Most patients with drusen and RPE changes alone do not develop significant vision loss. On the other hand, most patients progressing to the late-stage complications of geographic atrophy, exudative macular degeneration, or both will eventually develop moderate to severe central vision loss [13]. The prevalence of late-stage AMD in the US in 2019 was calculated to be 1.49 million people, which represents 7.5% of all the patients with AMD [5]. This difference between the prevalence of the non-vision-threatening early stage and vision-threatening late stage indicates that most patients will not develop moderate to severe central visual loss.

Macular degeneration is analogous to cardiovascular disease. Drusen and RPE changes are synonymous with plaque buildup on the coronary artery walls. As the drusen and RPE changes progress to intermediate and advanced stages, the risk of developing wet AMD or geographic atrophy increase [14]. In heart disease, advancing coronary artery plaque buildup results in coronary artery stenosis, which could lead to myocardial infarction, synonymous with wet macular degeneration, or ischemic cardiac heart failure, synonymous with geographic atrophy. Like cardiovascular disease, high cholesterol and coronary artery disease do not invariably lead to heart attack or cardiac heart failure. 

Development of drusen and RPE changes, like plaque buildup and high cholesterol, are insidious and asymptomatic. These signs are predominantly detected by physician examination or sophisticated imaging tools such as optical coherence tomography (OCT) [15]. The initial development of exudative macular degeneration or geographic atrophy are often symptomatically undetectable, like ischemic heart disease or silent myocardial infarction, and require medical examination or sophisticated imaging technology to detect, such as wide-field ophthalmoscopy [16]. Exudative AMD often presents with mild visual symptoms such as metamorphopsia, distorted vision, color change, contrast abnormality, or mild visual loss [17]. In GA, the location of atrophy determines the severity of vision loss, where central-involving GA will result in the recognition of a blind spot while non-central-involving GA will be asymptomatic [18,19].

### 1.3. Risk Factors

Risk factors that have been consistently associated with AMD are age, ethnicity, smoking, and genetic polymorphisms. Age is the most important risk factor for the development and progression of both early- and late-stage AMD. This importance is demonstrated by the low 3.5% prevalence of early and 0.1% for late AMD in those younger than 59 and the high 17.5% prevalence of early and 9.8% for late AMD in those older than 85. This difference is a 5 (early) and 98 (late) times increase in prevalence between these two age groups [20]. In regard to ethnicity, white Europeans have the highest annual incidence of both early and late AMD [21]. Smoking is the strongest associated modifiable risk factor in both early- and late-stage AMD patients [22].

In total, 103 AMD genes have been associated with AMD, but the most significantly associated are the complement factor H (CFH), age-related maculopathy susceptibility 2/high-temperature requirement A serine peptidase 1 (ARMS2/HTRA1), and apolipoprotein E (APOE) polymorphisms [23]. The most associated polymorphism to AMD is found in the CFH loci: the substitution of the histidine for tyrosine at the 42 codon of chromosome 1-region 31 (rs1061170), which results in the alteration of the sialic acid/heparin binding site in the short consensus repeat region 7 of the CFH protein [24,25,26]. Effectively, this polymorphism reduces binding with self-associated molecular patterns (SAMPs) and permits unchecked alternative complement activation and resultant chronic immune activation [27,28,29,30].

ARMS2/HTRA1 are genes that exhibit a high degree of linkage disequilibrium, so it is difficult to determine which polymorphism is responsible for its association with AMD [31]. ARMS2 is a protein that is expressed by human monocytes, binds to apoptotic cells, and recruits properdin, which facilitates C3b opsonization and phagocytosis of apoptotic and necrotic cells [32]. The polymorphism in ARMS2 reduces phagocytosis of necrotic and apoptotic debris and may result in accumulation of drusen material. The absence of ARMS2 can result in both reduced phagocytosis of apoptotic cells and increased nonspecific phagocytosis of healthy cells, leading to cellular loss like geographic atrophy [32].

HTRA1 polymorphism results in increased production of the HTRA1 protein, which is a serine peptidase known to cleave APOE [33]. Increased cleavage would inactivate APOE, mimicking the dysfunction brought about by the polymorphisms in APOE found in AMD. APOE’s best-known role is to regulate the transportation of lipids and cholesterol in the retina and brain [34]. Another major role is to protect lipids from complement attack by binding and activating CFH and protecting high-density lipoproteins (HDLs) from complement attack [35]. Polymorphisms in APOE associated with AMD also reduce APOE levels, dysregulating lipid and cholesterol clearance and inciting inflammation by not protecting the lipids from complement attack [35].

Major risk factors for AMD point to age-dependent innate immune overactivation as a major driver of disease development and progression into late-stage disease. Inflammation can also be detected clinically.

## 2. Central Role of Inflammation and Parainflammation in AMD

### 2.1. Clinical Evidence of Inflammation

In the early and intermediate stages of macular degeneration, measurable photoreceptor dysfunction is reflected in abnormalities dark adaptation, visual field, photo stress, and electro-retinographic changes [36,37,38,39]. These disease-correlated changes in visual function demonstrate inflammation as the underlying pathology behind all stages of macular degeneration. Prolonged dark adaptation, which progressively worsens in step with stage of AMD, is caused by inflammatory visual cycle impairment and indicates a worsening of inflammation as AMD progresses [40,41]. In one study, patients with early AMD had qualitative visual changes and symptoms of distortion that could be detected and quantified by visual field analysis [37]. These deficits of form recognition and sensitivity were found in areas of RPE atrophy not defined as geographic atrophy within this patient population, indicating asymptomatic vision loss in areas of overactive inflammation [37]. Photo stress recovery time, a measure of visual pigment recycling time, was inversely correlated with visual acuity, and inflammation-induced prolongation of recovery time was directly correlated with the presence or absence of geographic atrophy and advancing age [39]. In late AMD, inflammatory slowing of implicit time and amplitude reduction on electroretinograms (ERGs) of patients with geographic atrophy were seen in areas bordering fundus autofluorescence-defined geographic atrophy [42]. Foveal ERG performed in fellow eyes of patients with wet AMD who did not have severe visual acuity changes demonstrated implicit time prolongation, indicating that fellow eyes were also undergoing inflammation [38]. Visual cycle alteration represented the first indication of the central role of inflammation in AMD. 

### 2.2. Anti-Oxidant Therapy for Early-Stage AMD

While clinical findings in AMD pointed to inflammation as the central driver of AMD progression, initial therapeutics for AMD were focused on anti-oxidation. To this day, the only consensus treatment for early/intermediate-stage AMD is the use of anti-oxidant therapies studied in a large National Eye Institute study called the Age-Related Eye Disease Study 1 and 2 (AREDS 1 and 2) [43]. This study has produced over 28 reports and countless publications on the benefit of anti-oxidant therapy for the prevention of late AMD progression [44]. While the conclusions from these studies demonstrated a reduction in the rate of development of large drusen, geographic atrophy, and exudative AMD, the modest reduction in rate of progression implicates oxidation as only a stimulator of inflammation rather than the main driver for AMD progression [45,46].

### 2.3. Oxidation-Induced Dysfunctional Parainflammation in Early AMD

Demographic, environmental, and genomic risk factors combined with electrophysiological and psychophysical studies definitively implicate retinal inflammation as the major underlying factor in the development of all stages of AMD [47,48,49,50]. The degree of dysfunctional inflammation determines the stage and clinical presentation [51]. Multiple papers have described macular degeneration as a disease that is initiated by dysregulated parainflammation leading to the drusen stage of AMD then progressing to overt inflammation that triggers the late stage of AMD [52,53,54,55]. In 2008, Medzhitov postulated a parainflammatory state that lay between basal homeostatic conditions and true inflammation [56]. This parainflammatory state is considered an adaptive immune response to low level tissue stress such as the age-related accumulation of oxidative byproducts [57]. This acquired dysfunctional parainflammation that occurs with aging has been termed inflamaging and likely explains the early stages of AMD with drusen development and RPE changes [58]. The development of late exudative and geographic atrophy stages, on the other hand, is an overt innate immune activation with end-stage pathology determined by macrophage polarization [59].

**Figure 1:**
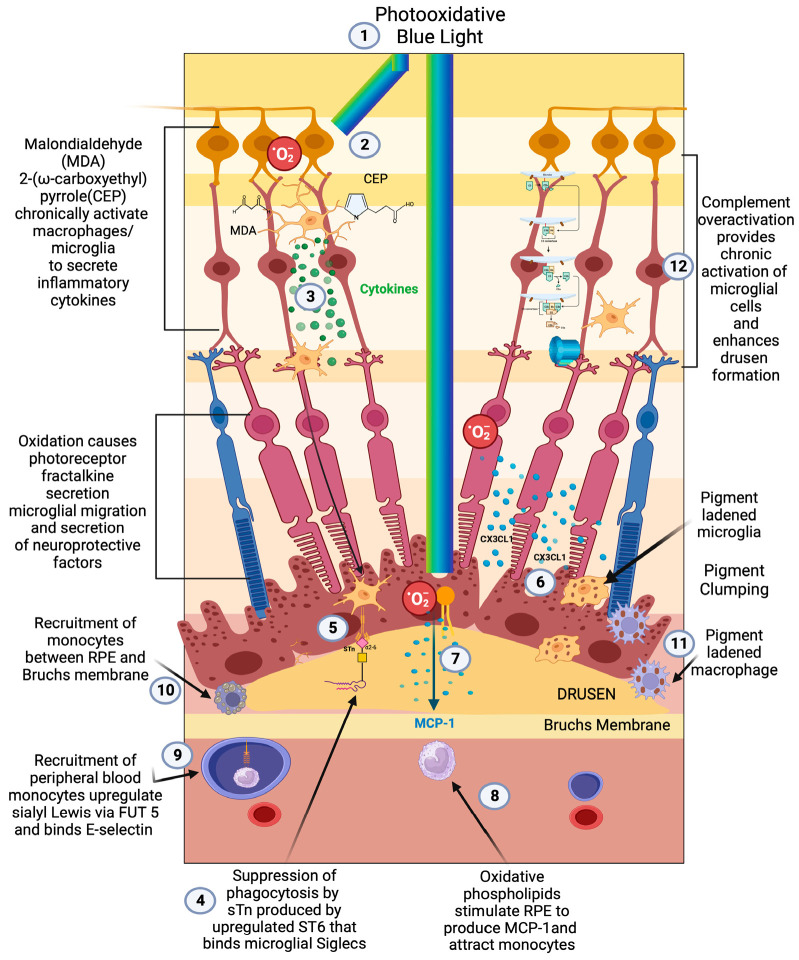
Early/intermediate-stage AMD. (1) Photo-oxidative blue light (2) oxidizes retinal lipids to produces oxidative byproducts (MDA, CEP) and reactive oxygen species, (3) which activate microglial cells to secrete cytokines. These activated macrophages are hindered from becoming phagocytic due to the (4) upregulation of ST6 that produces (5) sTN on the surface of retinal cells and drusen that agonizes Siglecs to prevent phagocytosis of drusen. (6) Reactive oxygen species also cause photoreceptors to secrete CX3CL1, which promotes migration of microglia and macrophages to the retina. (7) Oxidized phospholipids stimulate RPE cells to produce MCP—1 (8), which recruits peripheral blood monocytes (PBMCs) to areas of phospholipid oxidation. (9) Upregulation of FUT5 on PBMCs localizes monocytes to areas that are secreting chemokines such as CX3CL1 that upregulate e-selectin on vascular endothelium. (10) Monocytes are found between RPE cells and Bruch’s membrane. (11) Microglial cells and macrophages are also found to be pigment-ladened in this sub-RPE space and at the RPE cell layer, which appears as RPE pigment clumping. (12) The overactivation of complement caused by the polymorphism-induced impaired function of CFH will produce complement pathway metabolites that accumulate in drusen and can activate microglial cells.

Oxidative damage is the main tissue stress that stimulates parainflammation in the retina [60] (Figure 1(1)). The macula, which is exposed to photo, metabolic, phagocytic, and mitochondrial reactive oxygen species (ROS) production, is a site of tremendous oxidative stress. An association between blue light exposure (photo-oxidative light) and low anti-oxidant levels demonstrated an association with early and neovascular forms of AMD, implicating photo-oxidative light as a contributor to oxidative stress in AMD [61].

Photo-oxidative stress, which is focused and concentrated onto the macula by the ocular lens system [62], produces oxidative byproducts such as malondialdehyde (MDA) [63,64], 2-(ω-carboxyethyl)pyrrole(CEP) [65] (Figure 1(2)), and oxidized phospholipids (Figure 1(7)). CEP alone can activate microglia and initiate findings of early-stage AMD [66]. Oxidized phospholipids stimulate RPE to upregulate MCP-1m a major chemoattractant to macrophages [67] (Figure 1(8)). After photo-oxidative stress, fractalkine upregulation from the photoreceptor segment and outer nuclear layers mediates the cross talk between photoreceptors and microglia expressing the CX3CR1 receptor for fractalkines. This release of fractalkine serves to attract and activate microglia towards the injured photoreceptors [68] (Figure 1(6,7)).

Photoreceptors are constantly being bombarded with oxidative-, metabolic-, and toxic-mediated stress. Over exposure to photo-oxidative blue/violet light can cause S-cone retinal degeneration and apoptosis [62]. A fluorophore called lipofuscin is another source of ROS. Lipofuscin has been identified as Bis-retinoid N-retinyl-N-retinylidene ethanolamine (A2E), an autofluorescent toxic visual cycle metabolite that has been implicated in the progression of geographic atrophy [69]. A2E cannot be enzymatically degraded, and accumulations in RPE cells and in healthy youthful eyes are cleared by microglia, but those in elderly eyes are cleared by microglia and peripheral blood-derived macrophages [70]. When lipofuscin is exposed to blue/violet light, it produces more ROS, making it yet another source of oxidative stress [71].

RPE cells, due to their role in autophagy of the photoreceptor outer segment (POS), produce metabolically and phagocytic-activity-derived ROS [60]. RPE cells are also enriched with mitochondria to support their high metabolic activity, which in themselves are a major source of ROS [72]. RPE cells are the main housekeepers of the retina and are the most phagocytically active cells in the whole human body [73]. Because of the constant physiologic destruction of the POS from oxidative stress, the POS must be constantly shed and renewed. Through a process called heterophagy, RPE cells must ingest the shed POS and degrade them with their lysosomes [74]. Autophagy, a self-eating phenomenon, regulates oxidative stress as well as homeostasis of proteins, lipids, nucleic acid, and mitochondria and protects the cell from oxidized lipids, misfolded proteins, damaged organelles, and other cellular waste or faulty cellular components and directs them to the cellular lysosome [73]. Mitophagy, a specific form of autophagy, eliminates damaged or mutated mitochondria, which produce significant amounts of ROS [75]. Heterophagy, autophagy, and mitophagy are RPE cell functions that counterbalance the extreme oxidative environment and enable these cells to function as the main anti-oxidant protector of the retina [73,76]. Age-related impairment in these anti-oxidative RPE functions initiate parainflammation and early macular degeneration [77].

To enhance their anti-oxidative function, RPE cells also utilize nuclear factor erythroid 2 (Nrf2), a basic leucine transcription factor, to protect cells against oxidative stress by coordinating a transcriptional program that protects from oxidative cellular injury but allows cellular redox homeostasis [60]. In a non-oxidative environment, NRF2 is sequestered and degraded in the cytosol by Kelch-like ECH- associated protein 1 (Keap1), which is a substrate adaptor protein to a ubiquitin ligase complex, which prevents NRF2 from translocating into the nucleus. Interaction between Keap1 and ROS releases Nrf2 and translocates it into the nucleus, binding to the anti-oxidant response element (ARE). This binding results in the secretion of anti-oxidant enzymes like catalase and superoxide dismutase as well as regulating glutathione and thioredoxin levels [78]. An additional role for Nrf2 is to maintain mitochondria by controlling the expression of several mitochondrial enzymes that promote adenosine tri phosphate production (ATP) [79]. 

With age, the production of Nrf2 mRNA and protein decline along with the anti-oxidant response [80]. Aging also brings about decreased RPE phagocytosis, increased mitochondrial DNA damage, a decrease in mitophagy, reduced heterophagy, and dysfunctional autophagy [73]. This age-related impairment leads to accumulation of oxidative byproducts such as malondialdehyde (MDA) [63,64], 2-(ω-carboxyethyl)pyrrole(CEP) [65] (Figure 1(2)), and oxidized LDLs [81], which are stimulators of parainflammation and implicated in the initiation and progression of early AMD [66].

### 2.4. Complement Pathway-Induced Parainflammation

Despite the strong association of complement factor polymorphisms with the development of AMD and the development of two FDA-approved treatments for geographic atrophy (Pegcetacoplan and Avacincaptad pegol), there is a consensus that complement does not fully account for disease development [82,83,84,85]. Its direct role in disease may be in the development of drusen, and it may play only an indirect role in the development of late-stage disease [86] (Figure 1(12)). Evidence to support this role in producing drusen is found in the proteomic analysis of drusen from retinas of patients with AMD, where a large proportion of patients had complement proteins 9 (C9) and 3(C3) in their drusen [87]. C9 is a protein found in conjunction with the c5-9 complex or membrane attack complex. This finding agreed with earlier histopathologic analysis that corroborated the presence of multiple complement factors in drusen [88]. A meta-analysis found that systemic complement overactivation was a feature associated with early/intermediate AMD rather than late-stage geographic atrophy [89].

While no trials have been performed to determine the effect of complement factor depletion on the development of drusen, several trials have looked at the effect of complement C3(Pegcetacoplan) and C5 (Avacincaptad pegol) depletion and demonstrated a modest reduction in the growth rate of GA [90,91]. According to both pre-clinical and clinical studies, complement overactivation is important in initiating AMD by exacerbating parainflammatory overactivation and dysfunction, which enhances drusen formation. The inability of profound complement depletion to halt the progression of late-stage GA relegates complement as a minor player in the pathogenesis of late-stage AMD [90,91]. 

### 2.5. Early-Stage AMD Dysfunctional Parainflammation

At the cellular level, the early/intermediate stage is the accumulation of oxidized, metabolic, inflammatory debris that appears as yellow sub retinal deposits called drusen [87]. The pigmentary clumping seen in AMD represents pigment-ladened microglia or macrophage migration towards the retina, Bruch’s membrane, and under surface of the RPE [92] (Figure 1(9–11)). The accumulation of proinflammatory oxidative byproducts CEP and MDA leads to phagocytic/cytokine secreting microglial polarization, activation of parainflammatory mechanisms, and, potentially, recruitment of peripheral blood macrophages to the subretinal space to compensate for this accumulation of subretinal toxic substances [55] (Figure 1(2,3,10)). With age-dependent RPE cell senescence and dysfunction of autophagy, parainflammatory activation of microglial cells accelerates, resulting in the recruitment of peripheral blood-derived macrophages into the subretinal space [93]. 

### 2.6. Microglia’s Central Role in Dysfunctional Parainflammation

**Figure 2:**
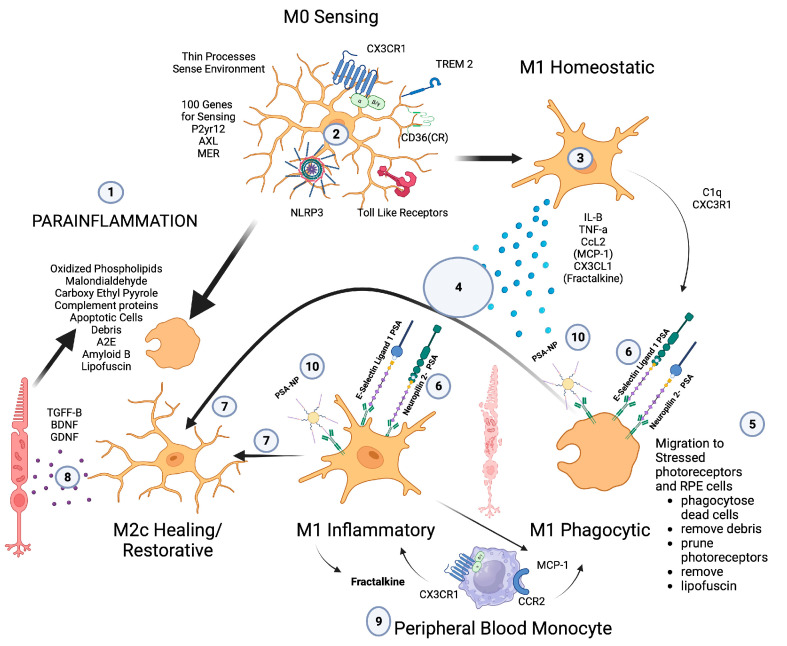
Microglial parainflammatory regulation: Microglia remove toxic metabolites, apoptotic cells, and oxidative debris while maintaining the health of photoreceptors and retinal cells in a process called parainflammation. (1) Parainflammation is initiated by pattern recognition receptors found on (2) M0 microglia such as TLR, TREM2, CX3CR1, NLRP3, and CD36, which use these receptors to bind and sense toxins, apoptotic cells, damage, and pathogen-associated molecular patterns. Once microglia are activated, they polarize to the (3) M1 like state, (4) secrete proinflammatory cytokines, and (5) migrate to the areas of stressed RPE cells and photoreceptors. (6) Upon activation, neuropilin-2-PSA and E-selectin ligand 1-PSA are secreted by these activated microglia. (6) The PSA on these glycoproteins binds Siglecs on activated microglial cells (7) to polarize them into the M2c healing state (8), which releases growth factors to protect and regenerate the stressed photoreceptors and RPE cells. (9) This homeostatic maintenance function of microglial parainflammation, if not adequately modulated with sialic acid checkpoint regulation, will result in recruitment of peripheral blood monocytes and AMD disease progression. (10) A PSA-nanoparticle (PSA-NP) mimics E-selectin ligand 1 PSA and Neuropilin 2-PSA to (7) polarize M1 activated microglia into the M2c healing state.

Microglia play a central role in modulating parainflammation [94] (Figure 2(1)). Microglial overactivation represents a common pathomechanism in a variety of retinal degenerative diseases and is often overactivated prior to the onset of overt retinal cell death [95]. In the retina, microglia’s dynamic motility allows comprehensive surveillance coverage of the entire retina in a short time period [96] (Figure 2(5)). This motility allows microglia to interact with retinal neurons and macroglia and play a central role in retinal homeostatic maintenance and clearance of cellular and metabolic debris [97]. 

The retinal environment is unique amongst all other CNS compartments in that it undergoes constant damaging stress, which in youth is maintained by RPE cells but with age and RPE cell senescence will lead to a constant microglial and parainflammatory activation state [54] (Figure 2(1)). To maintain this homeostatic retinal parainflammation, the regulators of microglial function have to be tightly controlled to determine the species of microglia that predominate. The different microglial states can be M0 sentinel and resting (Figure 2(2)), M1 proinflammatory and phagocytic, (Figure 2(3)), M2d anti-inflammatory and proangiogenic, M2a, b anti-inflammatory and profibrotic, and M2c anti-inflammatory, neuroprotective, and healing (Figure 2(7,8)). If the balance between these polarization states tilts towards any other states besides M2c, pathology will ensue. 

Activated microglia in the retina have been implicated in many retinal pathologies. In SD-OCTs, hyperreflective spots are visualized in AMD and diabetic retinopathy, which represent large amoeboid microglial cells, the activated microglial appearance, as opposed to the resting ramified microglial configuration [98,99]. Macrophages and microglia are bloated with engulfed membranous material near areas of drusen, suggesting that drusen components are restraining or checking phagocytic function [100,101]. While microglia can maintain retinal homeostasis during the first 5 to 6 decades of life, the onset of drusen and pigmentary changes signal the migration of microglia and the recruitment of peripheral blood-derived macrophages to Bruch’s membrane [102]. This sequence of events indicates the requirement of peripheral blood monocyte recruitment and loss of checkpoint restraint of these phagocytic cell in the progression of the drusen stage to the late stages of AMD.

### 2.7. Peripheral Blood-Derived Macrophages’ Role in Transition to Late AMD

The major role of peripheral blood monocyte-derived macrophages in the progression of the early drusen stage to late-stage geographic atrophy and exudative AMD is evidenced by the increase in number of activated macrophages in the choroid and Bruch’s membrane as AMD progresses. The observation that the highest number of activated macrophages are found in eyes with choroidal neovascularization further supports this central role [102].

The recruitment of peripheral blood-derived macrophages is a major function of retinal microglial cells when injury or toxic material overwhelm the microglia’s ability to phagocytose toxic oxidative byproducts, injured cells, or apoptotic cells [103]. To maintain macular health, the microglia must phagocytose oxidative byproducts (oxidized phospholipids, malondialdehyde, carboxyethyl pyrrole), apoptotic cells, complement proteins, abnormal proteins (Amyloid B), and toxic metabolites (A2E). If microglia do not encounter appropriate checkpoint ligands in the form of sialic acid, then chemokine signaling will recruit peripheral blood macrophages [104] (Figure 2(9)).

Patho-mechanistically, the recruitment of peripheral blood monocytes in clinically evident macular degeneration is mediated by microglial chemokine signaling, which is responsible for the recruitment of monocytes to the choroid and the retina. The chemokine receptors CCR2 and CX3CR1 and their respective ligands CCL2 (monocyte chemotactic protein-1, MCP-1) and CX3CL1 (fractalkine) mediate this recruitment [68,105,106].

While fractalkine-CX3CR1 signaling is proinflammatory, it is also critical for progesterone-mediated neuroprotection of the retina [107]. This dual role of Fractalkine-CX3CR1 interaction in microglial proinflammatory activation and neuroprotective properties demonstrates the necessity of tight regulation of microglial cells. If the proinflammatory properties of fractalkine-CX3CR1-activated microglia could be checked, but they could maintain their neuroprotective properties, then polarizing macrophages to the resolution state would eliminate inflammation and attenuate phagocytosis while providing neuroprotection. Without appropriate checkpoint regulation of microglial cells, the inflammatory activation state of microglia will promote more photoreceptor degeneration rather than rescue of photoreceptors.

### 2.8. Macrophage Recruitment Indicator of Late-Stage AMD

**Figure 3:**
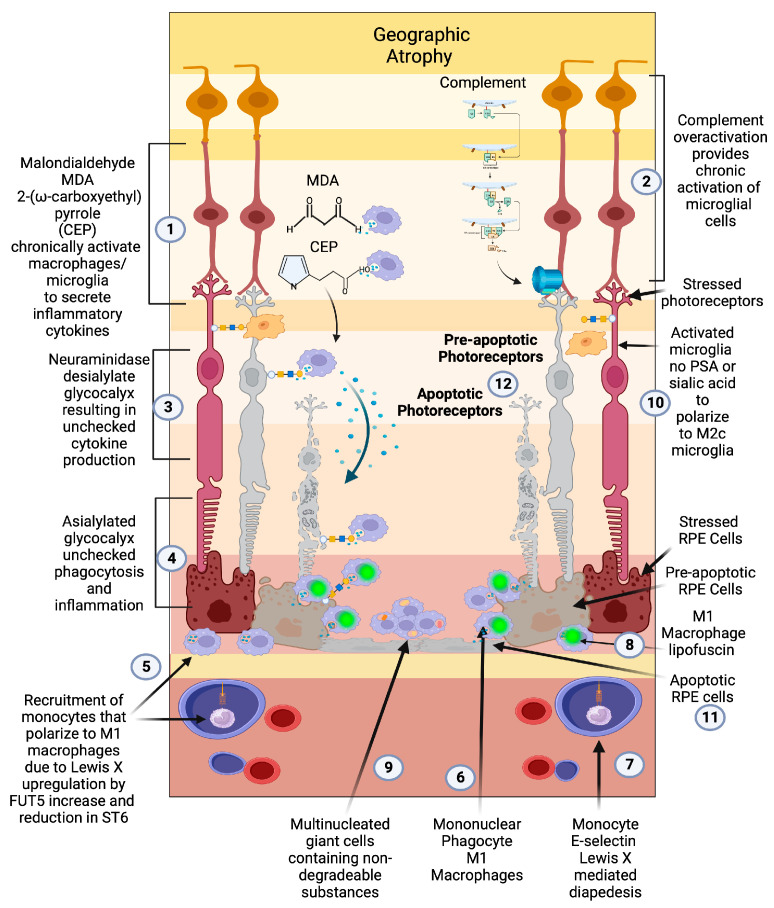
Geographic atrophy: (1) Progressive accumulation of oxidative byproducts (CEP, MDA) and (2) chronic overactivated complement pathway chronically activate microglia (3), which secrete neuraminidase and desialylate photoreceptors and RPE cells. (4) This loss of sialylation prevents restoration of homeostasis. (5) Chronically overactivated phagocytosis and inflammation recruits peripheral blood macrophages by upregulation of the fucosyltransferase FUT5 that produces Lewis X glycosylation on monocytes to bind E-selectin and (6) localize monocytes to sites of inflammation (7), allowing for diapedesis of the monocyte across the blood–retinal barrier. (8) These monocytes polarize to M1 macrophages when they enter the retina and are not able to clear substances like lipofuscin and other undegradable substances. (9) The macrophages form multinucleated giant cells because they phagocytose structures that are undegradable. (10) Since there is no sialic acid or polysialic acid to polarize to the healing M2c state, the macrophages are unchecked and result in elimination (11) first of the RPE cells then the (12) photoreceptors. The unchecked macrophages are the main determinant of growth of geographic atrophy.

The central role of macrophages in the pathogenesis of neovascular wet AMD is widely accepted [108,109,110,111], but the critical role of activated macrophages in the pathogenesis of geographic atrophy has been widely overlooked due to the focus on the complement pathway [112]. Prior to the genomic association between complement factors and AMD, histopathologic evidence pointed to macrophage/mononuclear phagocyte/multinucleated giant cells as the central causative factor in the pathogenesis of GA and the main phagocytic cause of retinal cellular clearance that manifests as RPE and photoreceptor loss [101,113] (Figure 3(5,6,9)).

One of the earliest demonstrations of macrophages’ central role was on electron microscopic/histopathologic studies of postmortem retinal specimens of seven patients with geographic atrophy [114]. In this study, mononuclear phagocyte series (MPS) cells (situated between the basement membrane of the RPE and inner collagenous layer of Bruch’s membrane) and multinucleated giant cells (derived from macrophage fusion created by inability to degrade constituents of Bruch’s membrane or RPE pigment) were found in or around areas of geographic atrophy [114]. Interestingly, no phagocytic RPE cells, which at that time were thought to be the cause of cellular loss, were found at the border or within GA. This hallmark study implicated microglia and macrophages as the main phagocytic cells in the pathology of geographic atrophy as opposed to RPE cells, the predominant phagocytic cell in the normal retina [73,101]. Clinico-pathologic correlation demonstrated that both MPS and giant cells containing pigment are typically found in the outer edge of the geographic atrophy lesion or in areas of coarse pigmentary mottling, which is clinically predictive of the development of geographic atrophy [115,116]. The close apposition of activated macrophages/microglial cells at the leading edge of GA indicates that it is monocyte-derived macrophages that phagocytose RPE and photoreceptors that lead to the development and growth of geographic atrophy in AMD [101].

The question that these findings raised in the 1990s was how the activation of macrophages resulted in the development of atrophic and neovascular phenotypes, which were considered two very different processes at the time. The answer was slowly revealed over the next two decades with the discovery of different macrophage/microglial polarization states [117]. The potential for macrophages/microglia to polarize between a phagocytic proinflammatory and a vascular endothelial growth factor-producing angiogenic polarization state determine the development of geographic atrophy, exudative AMD, or both [59].

### 2.9. Macrophage Polarization Determines Late-Stage AMD

The classically activated (M1) and alternatively activated (M2) binary description of macrophage polarization, based on biomarker expression and cytokine production, does not reflect the true character of these subtypes. A function-based description of macrophage polarization better characterizes their role in pathology [59,117,118]. The M1 polarization state is characterized as the proinflammatory phagocytic state. The M2 state can be subdivided into four M2 subtypes. The M2 a, b subtype are the anti-inflammatory pro-fibrotic type, the M2d is the pro angiogenic phenotype, and the M2c is the anti-inflammatory and neuroprotective type [117,118,119]. The M2c can also dedifferentiate myofibroblasts, so is considered anti-fibrotic [120].

In vitro, M1 macrophages are predominantly neurotoxic with modest axon growth-promoting effect, in contrast to the M2 macrophages, which promote long-distance axon growth without neurotoxicity [121]. In vivo studies in traumatic brain or spinal cord injury have characterized the time course and characteristics of M1/M2 polarization after injury [121,122].

M1-like macrophages release oxidative metabolites and proteases that kill neurons and glial cells [121]. In contrast, M2-like cells facilitate tissue repair [123]. In spinal cord injury models, increased M2c microglia expressed in the first week after injury correlated with better neurological outcome, indicating a healing neuroprotective function of M2c microglia/macrophages [122]. A time course study comparing M1 versus M2 levels in this model show that M1 expression is upregulated for at least a month, while M2 levels diminish drastically 1 week post injury. The level of M2a and M2c upregulation within the first week correlated well with neurological recovery. The neurological recovery resulted from the neuroprotective properties of microglia polarized to the M2c or M2a state. These properties were the secretion of trophic factors such as brain-derived neurotrophic factor (BDNF) and glial-derived neurotrophic factor (GDNF) and the ability to perform controlled phagocytosis, which prevents necrosis of surrounding tissue [124] (Figure 2(8)). 

**Figure 4:**
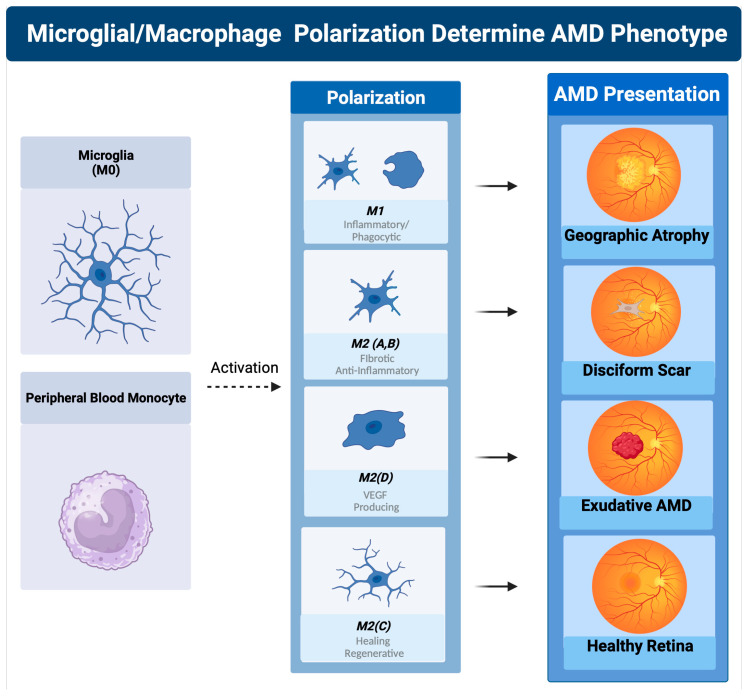
Macrophage polarization determines AMD phenotype. The plasticity and different polarization states correlate with the clinical picture seen in late-stage macular degeneration. In geographic atrophy, RPE cells and photoreceptors are phagocytosed as a function of the M1 polarized phagocytic macrophages. Exudative AMD is neovascularization produced by overexpression of VEGF, the main cytokine secreted by the M2d polarization state. The sequelae of exudative AMD untreated with anti-VEGF therapies is a disciform fibrotic scar in the control of the pro-fibrotic M2 A, B state. With appropriate sialic acid signaling such as PSA, all polarization states can transform into the healing M2c state.

While at first glance, macular degeneration may not appear to be a disease of acute CNS injury, the macrophage microglial behavior in the retina is consistent with what is seen in injury models of the CNS [55,125]. Eyes with advanced AMD had a higher ratio of M1 to M2 macrophages than age-matched normal autopsied eyes [126]. This enhanced chronic expression of neurotoxic phagocytic M1 macrophage with the reduction in M2c neuroprotective macrophages results in unchecked photoreceptor, RPE cell degeneration, and phagocytosis (geographic atrophy) [126]. The abundance of M1 polarized macrophages and adenosine polarize M1 to M2D VEGF-producing macrophages, resulting in the development of subretinal neovascularization [127]. If the M2 a, b polarization predominates, then retinal fibrosis will develop (disciform scar) [59]. Like CNS injury, the failure to polarize M1 macrophages towards the M2 c state will result in failure of functional recovery [122] (Figure 4). 

### 2.10. Sialylation Controls Microglial Activation and Macrophage Polarization in AMD

Non-genomic factors determine microglial activation and macrophage polarization in AMD. To determine which of these factors were important in AMD, Emilsson et al. performed a large proteo/genomic analysis of serum proteins in patients with macular degeneration [128]. This group identified several serum proteins that were elevated differentially in early and late AMD. As expected, serum protein complement factor H related 1 (CFHR1) correlated with early and late AMD. Complement factor H related 5 (CFHR5) correlated only with late-stage AMD. A novel but important finding from this study was the differential correlation of a class of proteins called sialyltransferase with different stages of AMD [128].

In this analysis, elevated serum protein levels of Alpha-N-acetylgalactosaminide alpha-2,6-sialyltransferase 1 (ST6GALNAC1/ST6) and Alpha-(1,3)-fucosyltransferase (FUT5) were highly correlated with patients with early-stage AMD [128]. In late-stage AMD patients, only FUT5 was found to be highly correlated. The expression of FUT5 in both late and early AMD and the expression of ST6 in only early AMD point to alterations of sialic acid glycan expression as a major determinant in the progression to late-stage AMD. It also implicates the absence of sialic acid self-associated molecular patterns (sSAMPs) in late AMD, which bind sialic acid-binding immunoglobulin-like lectins (Siglecs) to resolve activated macrophages to the resolution M2c state [129].

The loss of ST6 expression and the upregulation of FUT 5 in late AMD explain why microglia and macrophage activation is unchecked. When ST6 is expressed, the sialylated Tn antigen (sTn) rapidly sialylates the inflamed or oxidatively damaged glycocalyx of cells as well as the debris of these damaged cells [130]. This sialylation of the debris found in drusen [131] by sTN resolves activation of microglial cells, resulting in reduced phagocytosis, no recruitment of peripheral blood macrophages, no overt inflammation, and eventual accumulation of metabolic and inflammatory debris (Figure 1(9)). This debris, combined with complement pathway-created proteins and lipid byproducts, will accumulate in the subretinal space and worsen drusen and pigmentary clumping [88].

During the lates stage of AMD, ST6 is no longer upregulated; instead, FUT5 becomes the predominant sialyltransferase. FUT5 is a critical glycotransferase that produces Lewis x (3Galβ1,4[Fucα1,3] GlcNAc-), sialyl Lewis x (sLe^x^,1 NeuAcα2, 3Galβ1,4[Fucα1,3]GlcNAc-), Lewis a (Le^a^, 3Galβ1,3[Fucα1,4]GlcNAc), sialyl Lewis a (sLe^a^, NeuAcα2, 3Galβ1,3[Fucα1,4]GlcNAc-), and Lewis b (Le^b^, Fuc α1,2Galβ1,3[Fucα1,4]GlcNAc-). These glycans are the main binding determinants for selectins, in particular, e-selectin, which is responsible for localizing monocytes to areas of active inflammation [132]. Without sialic acid SAMP checkpoint restraint on activated microglia and recruited macrophages, immune cell activation will overproduce cytokines such as VEGF and phagocytose both drusen and underlying RPE cells and overlying photoreceptors, resulting in geographic atrophy (Figure 3(4,5)).

Clinical and proteomic findings in all stages of macular degeneration point to loss of microglia and macrophage sialic acid/Siglec immune checkpoint control as a major determinant of disease progression to late-stage geographic atrophy or CNV [133,134]. If appropriate sialic acid-mediated checkpoint control could be regained and homeostatic neuroprotective microglial function restored, late-stage disease could be halted and inflammation-impaired visual function could potentially be improved [135].

The alteration of the glycocalyx, “the sugar coat” of retinal cells, which in healthy young retinas are decorated with sialic acid caps (SAMPs), which agonize Siglec checkpoint receptors, determines the activation and polarization state of microglia and macrophages. By either binding to complement factors such as CFH and properdin or to Siglec receptors on immune cells, these sialic acid caps serve as checkpoint ligands to resolve innate immune activation [104]. This overlooked sialic acid sugar coating that determines the immune self is the master checkpoint regulator of immune cells and immune function [136].

Even in a non-inflammatory environment, the sugar coating on immune and host cells determines the polarization state of microglia and macrophages [137]. If there are enough sialic SAMP patterns, microglia are placed into the resolved state, which allows them to take on a neuroprotective and regenerative phenotype by producing cytokines such as BDNF or CTGF and selectively pruning neuronal dendrites and apoptotic cells [118,138]. In this sialic SAMP-checked state, microglia also can sense stressed, damaged, and sick cells or those undergoing apoptosis, which activate the microglia to the transient M1-like state to eliminate the damaged cell-released toxins and apoptotic cells [139]. If there are adequate sialic SAMPs available to bind the microglial Siglecs, the activation is short-lived and will result in the repolarizing of these M1 microglia to the M2c-like state [118].

Macrophage polarization determines how macular degeneration will clinically manifest, but it is the loss of cell surface sialic acid SAMP, a glycoimmune checkpoint ligand, that permits the disease to transition between the different polarization states [59,140]. Over time, overactivated complement and cumulative oxidative damage erodes the complex sialylated glycan structures of the cellular glycome [141]. Loss of sialic acid glyco-immune checkpoint restraint on microglial cells permits M1 activation of the microglia and active recruitment of peripheral blood macrophages. FUT5-dependent production of Lewis antigens drives diapedesis of peripheral blood monocytes by binding e-selectin on the inflamed vascular endothelium [132] (Figure 3(5,7)). The recruitment of predominantly M1 polarized macrophages to the retina in the absence of appropriate sialic acid SAMPs results in unchecked inflammation and clinical progression of macular degeneration. 

A potential therapy would be to agonize the Siglec and CFH checkpoint receptors with a sialic acid SAMP mimetic to attenuate complement amplification and repolarize activated microglia/macrophages to their non inflammatory, healing, and homeostatic form. This therapeutic strategy would normalize checkpoint control of the immune cell, in contrast to other anti-inflammatory strategies that deplete cytokines to inhibit inflammatory pathways [59,140].

## 3. Sialic Acid Agonism of Microglial/Macrophage Siglecs to Treat AMD

An ideal therapy for late-stage AMD such as GA would be to polarize M1 and M2d to the M2c state. Effectively, this strategy would attenuate both geographic atrophy and exudative AMD and promote photoreceptor survival by reducing proinflammatory mediators such as VEGF, attenuate pathologic phagocytosis, promote homeostatic phagocytosis, and secrete neuroprotective growth factors [55,129,142].

### 3.1. M2c Polarization: A Promising Therapeutic Strategy for AMD

As in spinal cord injury, elevating M2c results in better neuronal recovery [122]. A potential therapeutic strategy that could not only halt all forms of macular degeneration but restore visual function and retinal homeostatic health would be to reintroduce sialic acid control of microglia and macrophages. This strategy would increase M2c and decrease M1, M2d, and M2a microglia/macrophages by repolarizing them to M2c. If this repolarization strategy were to be possible, then not only would stopping progression of the disease be possible, but restoration of visual function and retinal health would also potentially be obtainable. 

### 3.2. Sialic Acid Can Repolarize Macrophages to M2c

Tumors’ ability to evade immune attack provides the blueprint for repolarizing microglia and macrophages to the healing M2c state. Many tumors hyper-express sialic acid by upregulating Golgi-resident sialyltransferases [143]. These sialyltransferases produce sialylation patterns on tumors that allow them to evade immune surveillance [144]. One well-characterized pancreatic ductal carcinoma-derived sialic acid pattern that binds Siglec 7 and 9 modulates monocyte/macrophages, reduces inflammatory signaling, increases PD-L1, a T-cell checkpoint ligand, and increases IL-10. This pancreatic cancer-produced sialic acid pattern agonizes Siglec-7 and 9 deactivates monocyte-derived macrophages to their quiescent M2c state, cloaking the tumor from both the innate and adaptive immune system and permitting tumors’ unchecked growth [145].

In fibrotic disease, serum amyloid protein (SAP), a well-characterized anti-fibrotic and anti-inflammatory protein, repolarizes fibrotic and inflammatory macrophages to the resolution M2c state [146]. The innate immune antifibrotic properties of SAP are mediated by the α(2,6)- linked terminally sialylated glycan found on the N32 position of SAP. If the sialic acid is removed from this glycan, it becomes proinflammatory and fibrotic [147]. C-reactive protein (CRP), which has similar sequence homology to SAP but is proinflammatory, can be made to behave like sialylated SAP by mutating CRP at position 32 from an alanine to an asparagine (CRP A32N). This mutation adds N-linked glycosylation to the surface of CRP. Functionally, CRP A32N behaves like SAP, enabling CRP A32N inhibition of fibrocyte differentiation in human peripheral blood monocytes [147]. 

### 3.3. Protein Sialic Acid Mimetics Not Feasible as Pharmaceuticals

This promise of developing immune-modulating therapeutic proteins with altered glycosylation is reduced by the difficulty and unpredictability of altering glycosylation by mutating amino acid sequences. When CRP A32N was mutated, only 40% of the recombinant CRP A32N was sialylated [147]. This absence of sialylation prevented the anti-fibrotic gain of function that sialylated CRP A32N produced. This failure can be explained by the post translational nature of cellular glycosylation. 

Protein glycosylation is based on O or N-linked glycosylation. O-glycosylation is defined by a sugar attachment to the oxygen atom on amino acids serine or threonine. N-linked glycosylation is defined by a sugar attachment to the nitrogen atom of an asparagine. To create a recombinant glycoprotein with the correct glycan expression pattern, the correct sialyltransferases must be upregulated to produce a particular glycan pattern. Since most recombinant proteins are produced in non-human cell lines, the cells do not express the correct glycosyltransferases. Because of these technological hurdles, these protein glyco-mimetic immune-modulating therapeutics are not druggable. 

### 3.4. Naked PolySialic Acid Not Feasible as Pharmaceuticals

The concept of just using sialic acid glycans by themselves has been investigated. In a laser-induced CNV model of exudative macular degeneration, α(2,8)-linked polysialic acid was able to reduce microglial/macrophage activation and recruitment, indicating its ability in the eye to agonize appropriate Siglecs, which in wild-type mice is Siglec-E, the mouse ortholog of Siglec 7 and 9 [148]. The intravitreal injection of this polysialic acid was also able to inhibit terminal complement complex formation and reduce the size and leakage of the neovascular lesions significantly [148].

Polysia was also shown to ameliorate inflammatory dopaminergic neurodegeneration in a lipopolysaccharide-induced Siglec 11 transgenic mouse model of Parkinson’s disease [149]. Intraperitoneal injection of LPS was followed by either injection of polysia or control, and brains were then examined for complement 4(C4) integrin alpha M (Itgam), a subunit of complement receptor 3 and C3. The brains were also probed for oxidative burst pathway enzymes such as nitric oxide synthase 2 (NOS2) and cytochrome b245 alpha and beta chain (Cyba/Cybb). The polysia only reduced C4 expression but not Itgam or C3 expression in the LPS-challenged mice. Polysia could not reduce Nos2 or Cyba [149]. It is unclear why PSA could not suppress these factors in vivo, but nonetheless, this does not support the use of PSA not presented on an NCAM-like protein as a therapeutic.

In a model of multiple sclerosis, polysia dp-24-30 reduced nitric oxide and recruited arginase-1-positive microglia to enhance remyelination in organotypic cerebellar slice culture of demyelination [150]. Interestingly, polysia of dp-8-14 reduced in vitro differentiation and did not help with remyelination. This observation demonstrates the specificity of Siglec receptors to the form of sialic acid and the presentation of the sialic acid [150].

Polysialic acid fragments have also been delivered through the intranasal route to the brains of mice who are deficient in polysia and in two mouse models of Alzheimer’s disease [151]. Alzheimer’s disease and schizophrenia have been associated with a deficit of neural cell adhesion molecule (NCAM), which is the brain’s main repository of polysialic acid. While mice receiving this treatment were shown to have rescued medial prefrontal cortex tasks, studies of intracranial pharmacokinetics demonstrated that these particles were only sparsely distributed in the mouse brain after 3 h and declined rapidly by 24 h [151].

While exogenous polysialic acid has demonstrated beneficial effects in animal models of wet AMD, Alzheimer’s, and Parkinson’s disease, the ability to optimize the sialic acid–Siglec immune cell synapse requires presentation, density, and persistence to mediate true immune cell deactivation [152]. If a therapy were to be developed for geographic atrophy, the drug would have to be administered monthly, so a drug would need to persist. This drug would also need to present sialic acids in a multivalent fashion. 

As has been demonstrated by these early proof-of-concept experiments, PSA alone would not be a feasible therapeutic from a biologic, pharmacokinetic, and biodistribution perspective. 

### 3.5. PSA-Nanoparticles Are Feasible Therapeutics for AMD

Nanoparticles decorated with sialic acid have been used to increase blood circulation time [153] and target tumor lectins for delivery of anti-tumor prodrugs [154]. The concept of decorating a nanoparticle with sialic acid to down-modulate inflammation by agonizing Siglecs receptors was first conceptualized and demonstrated in the laboratory of Professor Chris Scott. His lab decorated a nanoparticle with diasialic acid that functionally mimicked a protein covered with sialylated glycans. This diasialic acid α(2,8)-linked decorated nanoparticle was able to completely abrogate a model of sepsis and a model of acute respiratory distress [155]. This immune-modulating demonstration paved the way for a nanoparticle to be decorated with polysialic acid, targeting microglial cells and complement to abrogate microglial and macrophage M1 polarization by repolarizing macrophages to the M2c healing macrophage type [140].

This PSA-nanoparticle was designed to mimic PSA-bearing proteins such as Neuropilin-2 or E-selectin ligand -1. These PSA-bearing proteins are secreted by microglia as part of the negative feedback regulation of microglial inflammatory response to injury or toxic stress [156]. After LPS administration in cell culture, PSA-neuropilin-2 and PSA-E-selectin ligand 1 are released immediately in a metalloproteinase-dependent manner into the cell culture media for at least 24 h after LPS challenge. The release of microglial-derived polysia ligands that bind Siglec-E via a trans interaction, in the setting of acute brain injury, consolidates the role of polysialic acid ligands expressed on neuropilin-2 and e-selectin ligand 1 as the main agonistic activator of Siglec -E. This microglial activation-dependent Siglec-E agonistic pathway represents a negative feedback regulator of microglial activation, preventing unbridled inflammatory responses [157].

**Figure 5:**
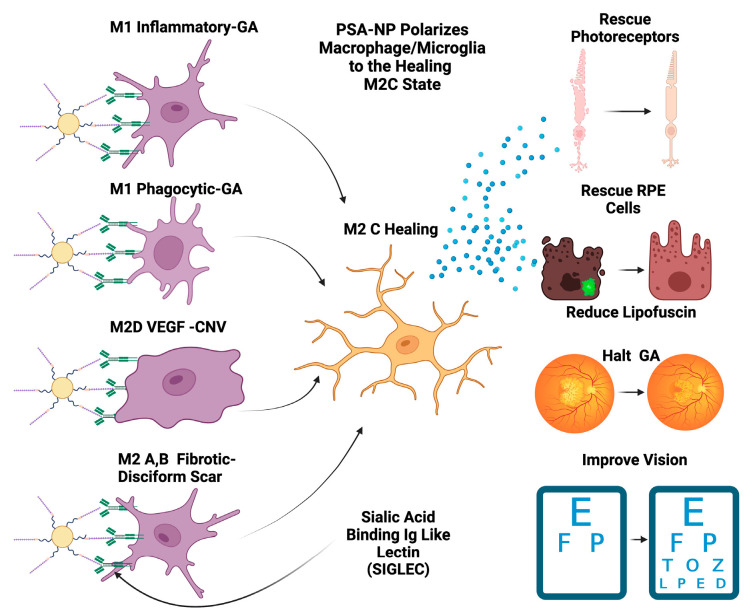
PSA-NP can halt GA and restore vision. PSA-NP can multivalently bind Siglecs on polarized macrophages and microglia, transforming them into the M2c healing state. These M2c healing macrophages/microglia will polarize M1 inflammatory, M1 phagocytic, M2D angiogenic, and M2a, b fibrotic M2c neuroprotective and BDNF- and GDNF-secreting macrophages. This in turn will rescue photoreceptors and RPE cells and restore the homeostatic parainflammatory function of microglia, potentially halting GA progression, reducing lipofuscin and improving visual function and vision.

If this nanoparticle can mimic PSA -neuropilin 2 or PSA-e-selectin ligand 1 and can repolarize activated M1 microglia/macrophages to the M2c healing state, this will decrease M1 and increase M2c. This repolarization has the potential to restore homeostatic healing microglial/macrophage function. In the setting of macular degeneration, this homeostatic restoration could theoretically cease cellular loss, normalize the parainflammatory environment, clear toxic substances such as autofluorescent lipofuscin, rescue pre-apoptotic photoreceptors/RPE cells, and restore efficient visual pigment regeneration. Potentially, a treatment such as this could halt geographic atrophy growth, reduce lipofuscin-mediated autofluorescence at the edge of the GA lesion, and recover visual function (Figure 5).

## 4. Future Directions

Therapeutic nanoparticle sialic acid SAMP-mimetic therapeutics are a novel technology that repolarize microglia and macrophage cells to their resolution healing state in a physiologic manner. This represents a paradigm shift in drug development for the treatment of geographic atrophy. Instead of targeting cytokines or complement factors that activate macrophages and microglia, these nanoparticle SAMP mimetics agonize Siglecs, the main autoimmunity preventing SAMP-recognizing checkpoint receptors. The promise of this therapeutic technology is its ability to mimic the cellular glycome to probe its interaction with both sialic acid-binding lectins and other lectin receptors that recognize sialylated or non-sialylated glycans. 

In total, 20 amino acids make up the building blocks of the proteome, four nucleotides make up the building blocks of the genome, and there are nine sugars that make up the glycome, which covers the surface of all cells in the human body [158]. These surface glycans are presented on cells in a multidimensional fashion that can form immune synapses with lectin receptor arrays to modulate the immune system. The ability to modulate immune cells by using poly-sialic acid-decorated nanoparticles to form a SAMP is just the beginning. Future research will:

- Present other novel end-linked sialic acid ligands on nanoparticles to characterize their ability to bind other Siglec receptors and to understand the in vitro and in vivo effects of these other receptor family members.
- Develop different sialic acid mimetic nanoparticles, specific to a single class of immune cells. This may lead to therapeutics for immune cell-specific driven diseases, filling a gap in unmet medical need.
- Use the eye as a model to characterize their effect on neuroinflammation and neurodegeneration that could be translated into therapeutics for diseases such as Alzheimer’s, multiple sclerosis, Parkinson’s, and stroke.
- Use the eye to characterize their immune-suppressive effect on both adaptive and innate immune systems that could be translated into therapeutics for diseases such as arthritis, vasculitis, allergies, and other conditions of inflammation.

## 5. Conclusions

Currently, a sialic acid nanoparticle has entered human clinical trials for the treatment of geographic atrophy secondary to age-related macular degeneration [140,159]. The PLGA nanoparticle core will be resistant to degradation and should demonstrate prolonged half-life in the vitreous [160]. The individual constituents of the nanoparticle are all biodegradable and have been proven safe for intravitreal injections [148,161]. Furthermore, this nanoparticle has demonstrably polarized M1 and M2a,b,d to the M2c phenotype in vitro and in vivo [140].

- The results of the clinical trial will determine if the pre-clinical demonstration of Siglec and CFH modulation by sialic acid agonism [140] can be translated into effective therapy for GA in AMD.
- If effective in GA, the therapy may be beneficial in other inflammatory retinal conditions such as diabetic retinopathy and uveitis. A multi-center clinical trial will commence in 2023 for the indication of diabetic macular edema.

## Data Availability

Data sharing is not applicable.

## Abbreviations

A2E	Bis-retinoid N-retinyl-N-retinylidene ethanolamine
AMD	Age-related macular degeneration
APOE	Apolipoprotein E
ARMS2	Age-related maculopathy susceptibility 2
AREDS	Age-Related Eye Disease Study
ARE	Anti-oxidant response element
ATP	Adenosine tri phosphate
BDNF	Brain-derived neurotrophic factor
C3	Complement factor 3
C4	Complement factor 4
C5	Complement factor 5
C5-9	Membrane attack complex
C9	Complement factor 9
CFH	Complement factor H
CFHR1	Complement factor H related 1
CFHR5	Complement factor H related 5
CCR2	Receptor for CCL2
CCL2	Monocyte chemotactic protein-1 (MCP-1)
CD36	Scavenger receptor recognizes oxidized phospholipids and lipoproteins
CEP	2-(ω-carboxyethyl) pyrrole
CNS	Central nervous system
CNV	Choroidal neovascularization
CRP	C-reactive protein
CTGF	Connective tissue growth factor
CX3CL1	Fractalkine
CX3CR1	Fractalkine receptor
Cyba	Cytochrome b245 Alpha
Cybb	Cytochrome b245 Beta
FUT5	Alpha-(1,3)-fucosyltransferase
GA	Geographic atrophy
GDNF	Glial-cell-derived neurotrophic factor
HTRA1	High-temperature requirement A serine peptidase 1
HDLs	High-density lipoproteins
Keap1	Kelch-like ECH- associated protein 1
LPS	Lipopolysaccharide
M0	Resting macrophage/microglia
M1	Proinflammatory/phagocytic/classically activated macrophage microglia
M2a	Anti-inflammatory/profibrotic/alternatively activated macrophage/microglia
M2b	Profibrotic/alternatively activated macrophage/microglia
M2c	Healing/resolution/anti-inflammatory/anti-fibrotic alternatively activated macrophage/microglia
M2d	Angiogenic/VEGF producing alternatively activated macrophage/microglia
MCP-1	Monocyte Chemotactic Protein-1
MDA	Malondialdehyde
MPS	Mononuclear phagocyte series
NCAM	Neural cell adhesion molecule
NLRP3	NOD-, LRR- and pyrin domain-containing protein 3
NOS2	Nitric oxide synthase 2
NRF2	Nuclear factor erythroid 2
PBMC	Peripheral blood-derived macrophage
PBMDMs	Peripheral blood monocytes-derived macrophages
PD-L1	Programmed death-ligand 1
POS	Photoreceptor outer segment
PSA	Polysialic acid
OCT	Optical coherence tomography
ROS	Reactive oxygen species
RPE	Retinal pigment epithelium
SAP	Serum amyloid P
sSAMP	Sialic acid self-associated molecular pattern
SAMP	Self-associated molecular pattern
Siglecs	Sialic acid-binding immunoglobulin-like lectins
ST6GALNAC1	Alpha-N-acetylgalactosaminide alpha-2,6-sialyltransferase 1
ST6	ST6GALNAC1
TGF-B	Transforming growth factor beta
TLR	Toll-like receptor
TREM2	Triggering receptor expressed on myeloid cells 2
VEGF	Vascular endothelial growth factor
